# Gravidity, parity and knee breadth at midlife: a population-based cohort study

**DOI:** 10.1038/s41598-022-16231-1

**Published:** 2022-07-20

**Authors:** Juho-Antti Junno, Asla Keisu, Maarit Niinimäki, Jaakko Niinimäki, Petri Lehenkari, Petteri Oura

**Affiliations:** 1 Medical Research Center, Oulu University Hospital and University of Oulu, Oulu, Finland; 2 Anatomy and Cell Biology, Cancer and Translational Medicine Research Unit, Faculty of Medicine, University of Oulu, P.O. Box 5000, 90014 Oulu, Finland; 3 Department of Archaeology, Faculty of Humanities, University of Oulu, Oulu, Finland; 4 Archaeology, Faculty of Arts, University of Helsinki, Helsinki, Finland; 5 Department of Obstetrics and Gynecology, PEDEGO Research Unit, Medical Research Center, Oulu University Hospital and University of Oulu, Oulu, Finland; 6 Department of Obstetrics and Gynecology, Oulu University Hospital, Oulu, Finland; 7 Research Unit of Medical Imaging, Physics and Technology, University of Oulu, Oulu, Finland; 8 Center for Life Course Health Research, Faculty of Medicine, University of Oulu, Oulu, Finland; 9 Department of Forensic Medicine, Faculty of Medicine, University of Helsinki, Helsinki, Finland; 10 Forensic Medicine Unit, Finnish Institute for Health and Welfare, Helsinki, Finland

**Keywords:** Musculoskeletal system, Reproductive signs and symptoms

## Abstract

Gestation increases the biomechanical loading of lower extremities. Gestational loading may influence anthropometrics of articular surfaces in similar means as bone diaphyseal properties. This study aimed to investigate whether gravidity (i.e. number of pregnancies) and parity (i.e. number of deliveries) is associated with knee breadth among middle-aged women. The study sample comprised 815 women from the Northern Finland Birth Cohort 1966. The median parity count of our sample was 2 and the median gravidity count 3. At the age of 46, questionnaires were used to enquire gravidity and parity, and posteroanterior knee radiographs were used to obtain two knee breadth parameters (tibial plateau mediolateral breadth (TPML) and femoral condylar mediolateral breadth (FCML)) as representatives of articular size. The associations of gravidity and parity with knee breadth were analyzed using general linear models with adjustments for height, weight, leisure-time physical activity, smoking, and education years. Individuals with osteoarthritic changes were excluded from our sample. The mean TPML in our sample was 70.3 mm and the mean FCML 71.6 mm respectively. In the fully adjusted models, gravidity and parity showed positive associations with knee breadth. Each pregnancy was associated with 0.11–0.14% larger knee breath (p < 0.05), and each delivery accounted for an increase of 0.20% in knee breadth (p < 0.01). Between-group comparisons showed that multiparous women had 0.68–1.01% larger knee breath than nulli- and primiparous women (p < 0.05). Pregnancies and deliveries seem to increase the mediolateral breadth of the knee. This increase is potentially associated with increased biomechanical loadings during gestation.

## Introduction

Gestation is associated with a physiological increase in body weight, changes in hormone status, and gait alterations^1–4^. Voerman et al.^5^ reported that the median gestational weight gain in their study population was 14.0 kg. Gestation and lactation periods as well as first postnatal years in general thus significantly increase the biomechanical loading on the weight-bearing articular surfaces of a female. Biomechanical load combined with hormonal changes and gait alterations potentially stimulate the knee joint to alter its shape, size, and other properties.

Studies on the skeletal effects of gravidity and parity have mostly addressed bone mineral density (BMD) at various skeletal sites, but have not been able to demonstrate long-term effects on maternal BMD^6,7^. However, gestation and lactation periods are found to be associated with short-term changes in bone mass^8^. These changes are often generated as the combined effect of biomechanical and hormonal alterations in the female body.

Generally, biomechanical loading has an effect on bone strength by influencing bone size and BMD (e.g. Ref.^9^). The importance of biomechanical loading for skeletal health is widely recognized (e.g. Ref.^10^). In light of the connection between loading, activity and bone geometry, weight-bearing joints could be assumed to be adaptable in a similar way to long bone shafts. However, factors that are associated with articular surface size are yet to be completely understood. In fact, articular surface size is commonly understood to remain relatively stable throughout adulthood (e.g. Ref.^11^). Major lifetime factors such as physical activity level seem to have minimal effect on joint size^12,13^. Apart from effects of pathological changes such as osteoarthritis, which affects women more often than men. Estrogen has a crucial effect on bone homeostasis by directly affecting both osteoclasts and osteoblast^14^. During pregnancy the placenta is the primary source of estrogen biosynthesis and the estrogen levels are high, but after delivery and during lactation estrogen levels fall considerably. In postmenopausal women the prevalence of osteoarthrosis increase, and the risk for knee replacement is higher among women with low estrogen levels^15^.

In the present study, we aimed to investigate whether gravidity (i.e. number of pregnancies) and parity (i.e. number of deliveries) were associated with knee size among middle-aged women. The material of the study was constituted by a large sample of Northern Finnish women at the age of 46 years. We hypothesized that gestational loading would potentially influence anthropometrics of articular surfaces by increasing the knee breadth. We believed that this specific sample could reveal the potential effects of gestation and lactation periods as it included a relatively high number of multiparous and grand multiparous women.

## Materials and methods

### Study population

This study utilized a subsample of prospective, population-based Northern Finland Birth Cohort 1966 (NFBC1966)^16^. Initially, NFBC1966 comprised pregnant women living in Northern Finland (i.e. the provinces of Oulu and Lapland) whose expected delivery dates fell between Jan 1 and Dec 31, 1966. The cohort included 12,068 mothers and 12,231 children, with a coverage of 96% of all births during 1966 in Northern Finland. Prospective data collection began in the 16th gestational week, and the NFBC1966 participants have been followed ever since. Broad questionnaires and clinical examinations have been used to gather information on the participants' health status and lifestyle habits.

At age of 46, a total of 5861 individuals responded to questionnaires and participated in clinical examinations. Of them, 1946 individuals residing in the Oulu region (100 km radius) underwent radiography of the knee joint. Of these, 1131 individuals were excluded due to (1) male sex, (2) missing reproductive or confounder data, (3) previous knee surgery, (4) bone pathologies in the radiographs (mostly osteoarthritic changes), or (5) technically inadequate radiographs. Thus, the final sample of this study comprised 815 women.

### Knee measurements

**Figure 1:**
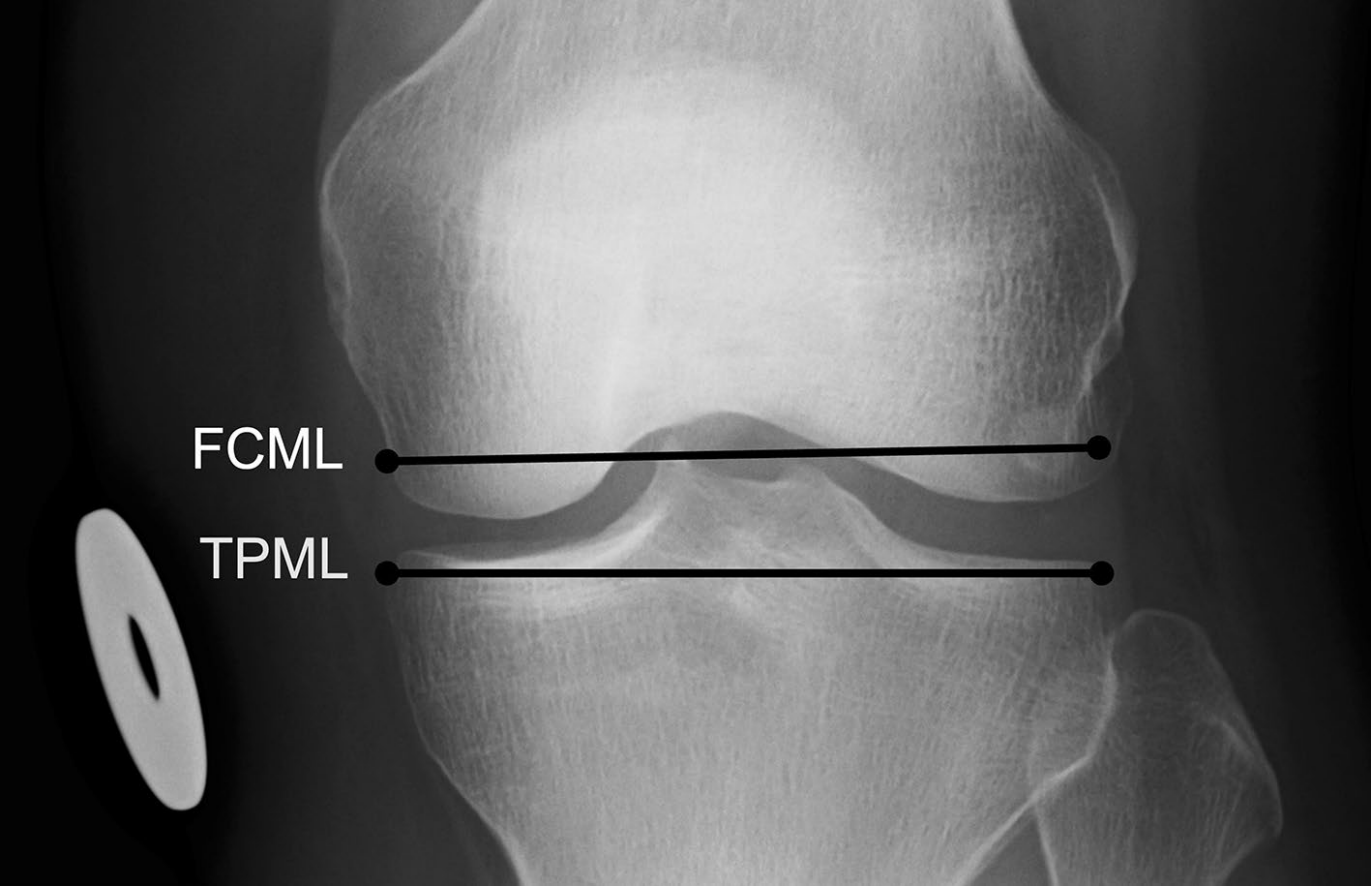
Knee measurements. *FCML* mediolateral breadth of femoral condyles, *TPML* mediolateral breadth of tibial plateau. Diameter of the calibration disc: 30 mm.

Knee breadth measurements were taken from digital radiographs of the right knee joint (Fig. 1) by an author of the study (A.K.). A detailed description of the procedure has been given in a previous publication^17^. Radiographs were accessed and measured using neaView Radiology software version 2.31 (Neagen Oy, Oulu, Finland). Posteroanterior radiographs were utilized, with individuals positioned in fixed flexion view^18,19^.

The following measurements were taken from each radiograph: (1) mediolateral breadth of the articular surface of the femoral condyles (FCML), and (2) mediolateral breadth of the articular surface of the tibial plateau (TPML). FCML was measured by drawing a line tangential to the inferiormost points of the femoral condyles; this line was transposed to the widest part between the femoral condyles. TPML was measured as close to the border of the tibial plateau as possible. Measurements were recorded to the nearest 0.1 mm. The initial measurements were converted into true sizes with the help of a metal calibration disc of 30 mm in diameter attached on the participant’s right leg. The technical error of measurement (TEM) and relative technical error of measurement (rTEM) were reported by Keisu et al.^17^, and the repeatability was high for all the measurements (TEM 0.1–0.5 mm, rTEM 0.1–0.6%).

### Reproductive history

In the 46-year follow-up questionnaire, women were asked about the number of deliveries, ectopic pregnancies, miscarriages and abortions they had undergone during their lifetime. As described in a previous publication^20^, gravidity was calculated as the overall number of pregnancies, and parity as the number of deliveries of each woman. Women with no deliveries were classed as ’nulliparous’, those with one delivery as ’primiparous’, and those with a history of several deliveries were classed as ‘multiparous’. Those with ≥ 5 deliveries were classed as ‘grand multiparous’.

### Confounders

In the clinical examination at the age of 46, a study nurse systematically measured the height and weight of each individual. Body mass index (BMI) in kg/m^2^ was calculated as weight divided by height squared.

Education, smoking history and leisure-time physical activity were elicited in the 46-year follow-up questionnaire. Education years, a proxy for socioeconomic status, was determined by asking: ‘What is your basic education? (1) Less than 9 years of elementary school, (2) elementary school, (3) matriculation examination’; and What is your vocational education? (1) None, (2) occupational course, (3) vocational school, (4) vocational college, (5) polytechnic, (6) university, (7) other, (8) unfinished course’. The responses were classed according to the Finnish education system as follows: < 9 years, 9–12 years, or > 12 years.

Smoking history was elicited using two questions: (1) ‘Have you ever smoked cigarettes (yes/no)?’ and (2) ‘Do you currently smoke (yes/no)?’. Individuals were classed as non-smokers, former smokers, or current smokers.

Leisure-time physical activity was elicited by asking: ‘How often do you participate in brisk physical activity/exercise [defined as causing at least some sweating and breathlessness] during your leisure time? (1) Daily, (2) 4–6 times a week, (3) 2–3 times a week, (4) Once a week, (5) 2–3 times a month, (6) Once a month or less often. The responses were regrouped as follows: < 1 times/week, 1–3 times/week, or ≥ 4 times/week.

### Statistical analysis

SPSS software (IBM, Armonk, NY, USA) version 27, 64-bit edition was used for the statistical analyses. P values < 0.05 were considered statistically significant. Means with standard deviations (SDs), medians with interquartile ranges (IQRs) and percentages with frequencies were used as descriptive statistics. Characteristics of the sample were presented before and after stratification by parity.

The associations of gravidity and parity with knee breadth (i.e. FCML and TPML in mm) were analyzed using general linear models. Beta coefficients, 95% confidence intervals (CIs) and P values were documented from the output. Models were first constructed without adjustments (unadjusted models), and then confounder variables were added (adjusted models). Gravidity and parity were modelled as continuous variables (where beta coefficients are interpreted relative to one pregnancy/delivery), and by comparing groups with each other (e.g. multiparous vs. others, where beta coefficients represent mean difference between groups).

### Ethical considerations

The study adhered to the principles of the Declaration of Helsinki, with voluntary participation and signed informed consent. Sensitive details were replaced by anonymous identification codes. The Ethics Committee of the Northern Ostrobothnia Hospital District approved the study.

## Results

The sample consisted of 815 women whose characteristics are presented in Table 1. Most women were never-smokers, physically active several times per week, and had attended school 9–12 years. As regards reproductive history, most women had delivered at least twice, and the sample included 47 women who had delivered ≥ 5 times. Of the knee breadth parameters, mean FMCL was 71.6 mm among the full sample (71.7, 71.4, 71.7, and 72.2 mm among nulliparous, primiparous, multiparous and grand multiparous women, respectively). Mean TPML was 70.3 mm among the full sample (70.2, 69.8, 70.4, and 70.8 mm among nulliparous, primiparous, multiparous and grand multiparous women, respectively).

**Table 1:** Main characteristics of the study population.

	All women (n = 815)		Stratified by parity							
			Nulliparous (n = 115)		Primiparous (n = 119)		Multiparous (n = 581)		Grand multiparous (n = 47)	
**Anthropometry**										
Height^a^ (cm)	164.8	5.7	165.9	6.1	165.1	6.1	164.5	5.5	165.0	5.0
Weight^a^ (kg)	69.7	12.4	69.7	14.0	69.8	11.6	69.6	12.2	71.5	12.1
Body mass index^a^ (kg/m^2^)	25.7	4.4	25.4	5.1	25.6	4.2	25.7	4.4	26.4	5.0
**Leisure-time physical activity**										
< 1 time/week^b^	24.2	197	20.0	23	26.1	31	24.6	143	21.3	10
1–3 times/week^b^	59.0	481	56.5	65	57.1	68	59.9	348	66.0	31
≥ 4 times/week^b^	16.8	137	23.5	27	16.8	20	15.5	90	12.8	6
**Smoking history**										
Never-smoker^b^	59.1	482	60.9	70	47.9	57	61.1	355	74.5	35
Former smoker^b^	24.2	197	17.4	20	30.3	36	24.3	141	14.9	7
Current smoker^b^	16.7	136	21.7	25	21.8	26	14.6	85	10.6	5
**Educational history**										
< 9 years^b^	2.7	22	1.7	2	2.5	3	2.9	17	8.5	4
9–12 years^b^	69.1	563	66.1	76	75.6	90	68.3	397	66.0	31
> 12 years^b^	28.2	230	32.2	37	21.8	26	28.7	167	25.5	12
**Knee breadth**										
Femoral condylar mediolateral breadth^a^ (mm)	71.6	3.3	71.7	3.4	71.4	3.7	71.7	3.3	72.2	3.1
Tibial plateau mediolateral breadth^a^ (mm)	70.3	3.1	70.2	3.3	69.8	3.5	70.4	3.0	70.8	2.6
**Reproductive history**										
Gravidity count^c^	3	2–4	0	0–1	1	1–2	3	2–4	9	6–11
Parity count^c^	2	1–3	0	0–0	1	1–1	2	2–3	7	5–9
Nulliparous^b^	14.1	115	–	–	–	–	–	–	–	–
Primiparous^b^	14.6	119	–	–	–	–	–	–	–	–
Multiparous^b^	71.3	581	–	–	–	–	–	–	–	–
Grand multiparous^b^	5.8	47	–	–	–	–	–	–	–	–

^a^Values are means with standard deviations.

^b^Values are percentages with frequencies.

^c^Values are medians with interquartile ranges.

**Figure 2:**
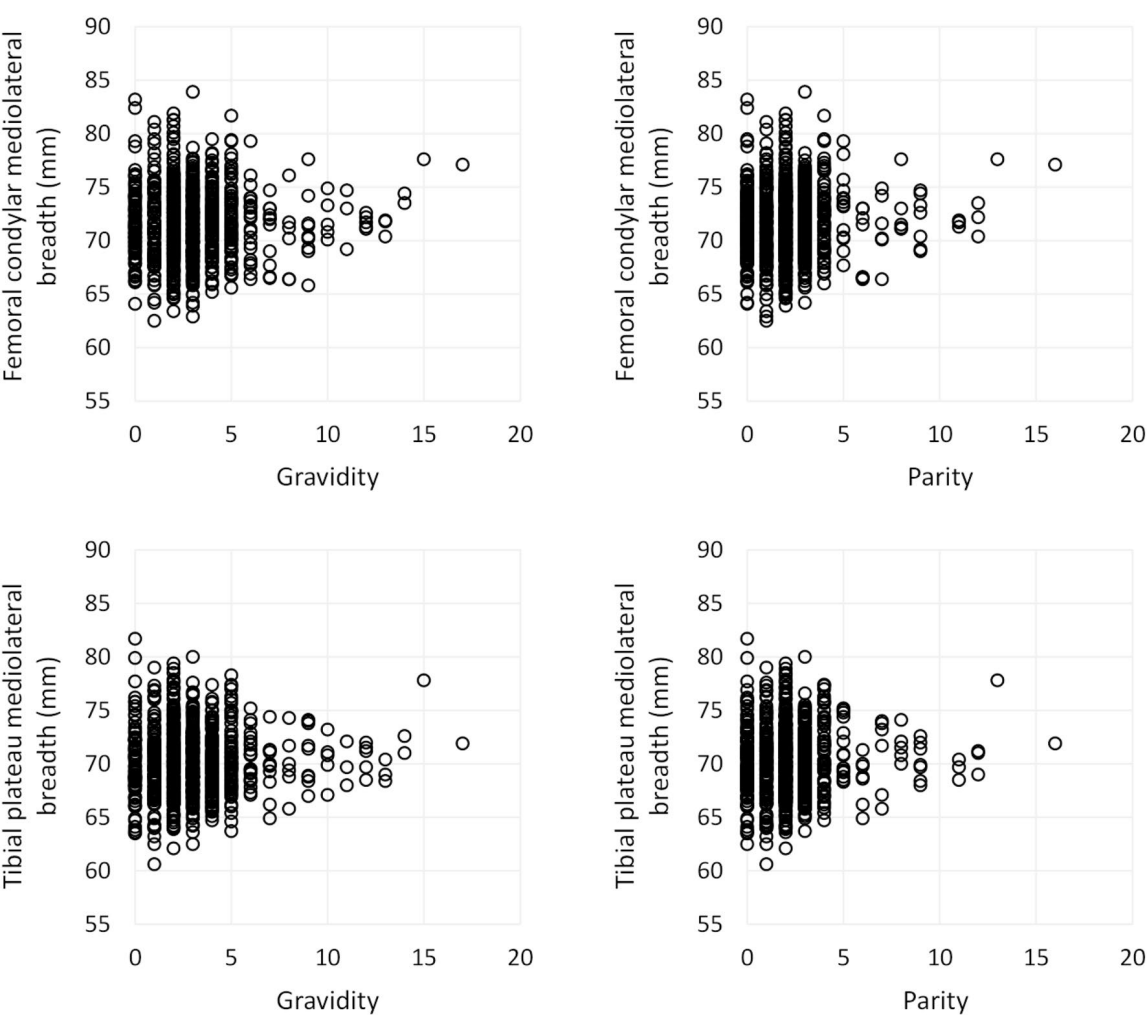
Scatter plots demonstrating the association between gravidity, parity, and knee breadth measurements.

The associations of gravidity and parity with knee breadth are shown in Table 2. Scatter plots are shown in Fig. 2. The unadjusted analyses did not show significant associations between gravidity, parity and knee breadth. However, after incorporating adjustments in the models, gravidity and parity showed positive associations with knee breadth. When modelled linearly, each pregnancy was associated with 0.11–0.14% or 0.08–0.10 mm larger knee breath (p < 0.05), and each delivery accounted for an increase of 0.20% or 0.14 mm in knee breadth (p < 0.01). Between-group comparisons showed that multiparous women had 0.68–1.01% or 0.49–0.71 mm larger knee breath than nulli- and primiparous women (p < 0.05).

**Table 2:** Association between gravidity, parity and knee breadth dimensions among the study population. Beta coefficients from general linear models.

Predictor	Outcome: FCML (in mm)				Outcome: TPML (in mm)			
	Unadjusted beta (95% CI)	P	Adjusted beta^a^ (95% CI)	P	Unadjusted beta (95% CI)	P	Adjusted beta^a^ (95% CI)	P
Gravidity count^b^	0.05 (− 0.06; 0.15)	0.374	**0.08 (0.01; 0.17)**	**0.045**	0.06 (− 0.03; 0.16)	0.199	**0.10 (0.02; 0.18)**	**0.014**
Parity count^b^	0.10 (− 0.02; 0.23)	0.104	**0.14 (0.04; 0.24)**	**0.008**	0.11 (− 0.01; 0.23)	0.060	**0.14 (0.04; 0.24)**	**0.005**
Multiparous vs. others^c^	0.13 (− 0.37; 0.64)	0.605	**0.49 (0.08; 0.91)**	**0.021**	0.40 (− 0.07; 0.88)	0.098	**0.71 (0.32; 1.10)**	**< 0.001**
Grand multiparous vs. others^c^	0.61 (− 0.37; 1.59)	0.224	0.53 (− 0.28; 1.34)	0.200	0.50 (− 0.43; 1.42)	0.290	0.40 (− 0.37; 1.17)	0.313
Multiparous vs. nulliparous^c^	− 0.04 (− 0.69; 0.62)	0.913	0.44 (− 0.11; 0.99)	0.119	0.18 (− 0.44; 0.79)	0.570	**0.61 (0.09; 1.13)**	**0.022**
Grand multiparous vs. nulliparous^c^	0.50 (− 0.62; 1.62)	0.380	**1.04 (0.04; 2.03)**	**0.041**	0.53 (− 0.55; 1.61)	0.332	0.91 (− 0.07; 1.88)	0.068

Significant values are in bold.

*CI* Confidence interval, *FCML* Femoral condylar mediolateral breadth, *P* P value, *TPML* Tibial plateau mediolateral breadth, *Vs.* versus.

^a^Adjusted for height, weight, leisure-time physical activity, smoking, and education years.

^b^Modelled linearly as a continuous variable. Beta coefficients are interpreted relative to one pregnancy/delivery.

^c^Modelled as a binary variable. Beta coefficients represent mean difference between groups.

## Discussion

Utilizing a population-based middle-aged cohort sample, we investigated whether gravidity and parity were associated with knee breadth. We detected a clear positive association between gravidity, parity and knee breadth, confirming our initial hypothesis. We believe that this is the first population-based study to address the association between reproductive history and knee dimensions. Our results suggest that obstetric factors such as increasing gestational load, gait alterations or hormonal influences may affect knee breadth. Other factors such as knee pathologies may also increase joint dimensions, However in this study we excluded Individuals with osteoarthritis or other clear knee pathologies from our sample. The present work was a cross-sectional study and thus only enabled us to investigate the association between reproductive history and knee breadth.

Gestation and lactation have clear effects on bone metabolism and structure^21^. During pregnancy the requirement for calcium intake is increased due to skeletal development of the fetus^22^. In lactation, additional calcium intake is necessary to produce breastmilk. According to recent meta-analysis, BMD is reduced during pregnancy and lactation, especially if the breastfeeding exceeds 6 months^23^. However, most clinical and animal studies suggest that they have no long-term effects on bone strength^24^. Studies about the effects of obstetric load on bone geometric properties in particular are sparse. Most of these studies are based on animal models (e.g. Ref.^25^) and demonstrate decrease of the bone strength during lactation.

Although parity has been positively associated with lumbar lordosis^26^, our previous study among Northern Finnish women suggested that obstetric factors such as increased and altered biomechanical loads or hormonal factors did not seem to affect vertebral geometry^20^. As such, the present association between gravidity, parity and knee breadth was clearly distinct in light of the previous findings. Potentially, as knee joint supports most of the body weight, the effects of biomechanical loading during gestation and lactation periods are more pronounced in the knee than the lumbar spine. Our finding could also be associated with the observed sex differences in the alignment of lower extremities^27^.

In our study, all significant findings concerned the adjusted models, as adjustments appeared to augment effect sizes. This implies that the unadjusted models were subject to confounding effects. For example, obesity is known to negatively influence fertility but may also lead to larger bone size due to increased loading.

The main strength of this study was the relatively large sample size of 815 women. General characteristics of the sample indicate that they represent the typical Northern Finnish population^20,28^. Importantly, a clear majority of the women had delivered at least twice, and up to 5% were grand multiparous. Other strengths were the homogeneous age as all were born in 1966 and thus secular trends did not confound our results. We also excluded individuals with pathologies in the knee and measured the knee dimensions from the radiographs with high reliability and low measurement error.

The study had several limitations. As the radiographs were taken in one timepoint at 46 years of age, we were unable to study the potential longitudinal change in knee breadth. The confounding effects of duration of lactation, hormonal factors, infertility or other comorbidities could not be fully ruled out. Also, we did not have data on menopausal status or use of hormone replacement therapy (HRT) of the participants, but in the previous study 16% of women in Northern Finland Birth Cohort were menopausal or late perimenopausal and 22% of them (3% of the whole female cohort) were using HRT^29^. These women were thus a minor group. No hormone measurements were available in the analyses, but knowing the average duration of pregnancy is 40 weeks, we can estimate that every pregnancy ending to delivery exposes women to excessive estrogen exposure, weight gain and changes in gait.

We conclude that gravidity and parity are associated with knee breadth among middle-aged women. Our findings suggest that gestation contributes to larger knee joint size, potentially via increased biomechanical loading. Future studies are needed to confirm the present findings, and to further characterize the roles of biomechanical, hormonal and gait changes during gestation and lactation in the context of skeletal response.

## Data Availability

The datasets generated and/or analysed during the current study are not publicly available due to confidential and ethical reasons but are available from the corresponding author on reasonable request.
